# Biomarkers associated with cognitive impairment in post-traumatic stress disorder: A systematic review of current evidence^☆^

**DOI:** 10.1016/j.arr.2024.102198

**Published:** 2024-03

**Authors:** Junling Guo, Vasiliki Orgeta, Isadora Olivé, Erik Hoff, Jonathan Huntley, Miranda Olff, Sjacko Sobczak

**Affiliations:** a Division of Psychiatry, University College London, London, United Kingdom; b Department of Neurology, Zuyderland Medical Center, Heerlen, Sittard, the Netherlands; c Department of Psychiatry, Amsterdam University Medical Center, University of Amsterdam, Amsterdam Neuroscience, & Amsterdam Public Health, Amsterdam, the Netherlands; d ARQ National Psychotrauma Centre, Diemen, the Netherlands; e Department of Neuropsychology and Psychopharmacology, Faculty of Psychology and Neuroscience, Maastricht University, Maastricht, the Netherlands; f Mondriaan Mental Health Center, Heerlen, Maastricht, the Netherlands; g Rotterdam University of Applied Sciences (RUAS), Research Center Innovations in Care, Rotterdam, the Netherlands

**Keywords:** Cognitive impairment, Dementia, PTSD, Biomarkers, MRI, PET, Amyloid, tau

## Abstract

**Objective:**

This systematic review aimed at synthesizing current evidence on biomarkers associated with cognitive impairment (CI) in Post-Traumatic Stress Disorder (PTSD).

**Methods:**

A systematic literature search was conducted for studies assessing biomarkers associated with CI in PTSD.

**Results:**

Of the 10,149 titles screened, 8 studies met our inclusion criteria. In a single longitudinal study, MRI volumes, Aβ and tau accumulation were not associated with CI in PTSD. Studies on structural imaging reported no significant association between morphological changes and CI. Two studies on diffusion neuroimaging showed abnormalities in white matter tracts which were cross-sectionally associated with CI in PTSD. Similarly, lower resting-state functional connectivity in neocortical networks, and elevated tau in the neocortex were also cross sectionally associated with CI. Two single studies on biochemical biomarkers showed that sixteen novel plasma proteins and lower BDNF, indicative of genetic vulnerabilities associated with neural and synaptic dysfunctions commonly observed in neurodegeneration, were cross-sectionally associated with CI in PTSD. Overall, evidence is of low quality.

**Conclusions:**

Longitudinal research utilizing large representative samples of trauma exposed populations are needed to establish the utility of specific biomarkers in monitoring cognitive decline in PTSD.

## Highlights

• A systematic review on biomarkers of cognitive impairment (CI) in PTSD was conducted; 8 studies met inclusion criteria.
• Despite their potential importance, no biomarker has as yet been validated.
• In a single longitudinal study identified, MRI volumes, Aβ and tau accumulation were not associated with CI in PTSD.
• Diffusion neuroimaging showed abnormalities in white matter tracts which were cross-sectionally associated with CI in PTSD.
• This paper provides clear recommendations on how to overcome the current issues in this research field.

## 1 Introduction

Post-traumatic stress disorder (PTSD) is a prevalent psychiatric disorder which usually develops after exposure to a traumatic event such as personal assault, a severe accident, or other threatening event (Bryant, 2019, Friedman et al., 2011, Kessler et al., 1995). It is characterized by persistent re-experiencing intrusive distressing memories associated with the life threatening event, avoidance, hyperarousal and dissociative symptoms (Hayes et al., 2012). Epidemiological studies estimate that around 13.0–20.4% of women and 6.2–8.2% of men are affected by PTSD (Breslau et al., 1991, Kessler et al., 1995, Olff, 2017), with people with dementia experiencing a high comorbidity rate (Sobczak et al., 2021). A recent systematic review and meta-analysis showed that PTSD increases risk of future dementia (Gunak et al., 2020), with both civilians and veterans with PTSD being at higher risk of developing cognitive impairment (CI) and dementia (Gradus et al., 2019, Roughead et al., 2017).

Cognitive impairment in PTSD is an heterogeneous entity that covers several forms of cognitive impairment in function of the number and type of cognitive domains affected (Scott et al., 2015). Although the mechanisms that may mediate the association between PTSD and future dementia risk remain largely unknown, several genetic, neuro-biological, and lifestyle factors have been proposed that may explain this association (Desmarais et al., 2020, Friedman et al., 2019). These include the vulnerability of the hippocampal network being susceptible to age and dementia-related neurodegeneration (West, 1993), specific epigenetic changes (i.e. DNA methylation) linking PTSD and AD such as accelerated aging and stress-related neurotoxicity (Antonelli-Salgado et al., 2021, Lohr et al., 2015), and several genetic candidates associated with PTSD and CI compared to control populations (Marchese et al., 2022). Epidemiological data have shown that PTSD is associated with increased rates of comorbidities linked to immune dysregulation such as metabolic syndrome, atherosclerotic cardiovascular disease, and autoimmune diseases, which may contribute to neuronal loss and reduced plasticity in key brain regions (Hori and Kim, 2019). PTSD may also promote oxidative stress which may in turn accelerate cellular aging and neurodegeneration (Miller and Sadeh, 2014).

It is not however currently known which subgroups of people with PTSD confer a higher risk of progression to cognitive decline and dementia. In this sense, biomarkers that can predict future outcome could be useful to longitudinally track the underlying disease pathology in an objective way. For example, identification of ‘cognitive impairment risk’ biomarkers in PTSD may inform the development of targeted interventions for people at risk of experiencing cognitive decline and implementing preventative strategies that may delay neurodegeneration and further cognitive decline into dementia. The aim of our present study therefore was to systematically report on current knowledge on biomarkers associated with CI in PTSD and make recommendations for their use. A secondary objective was to assess the methodological quality of studies to date to inform future research in the area.

## 2 Methods

This systematic review was prospectively registered in PROSPERO International Prospective Register of Systematic Reviews (PROSPERO identifier CRD42023448944) and performed according to the Transparent Reporting of Systematic Reviews and Meta-analyses (PRISMA) Statement (Page et al., 2021).

### 2.1 Search strategy

We searched Medline Ovid, PsycINFO, and Embase up to January 2023 for studies that included biomarkers that were associated with CI in PTSD, using terms for 1) cognitive impairment or dementia, 2) “neurobiological markers”, and 3) “post-traumatic stress disorder” and other relevant search terms. The complete search strategy is provided in Supplementary Materials. In line with the NIH Biomarker Working Group definition (Group, 2016), we considered as biomarker “a defined characteristic that is measured as an indicator of normal biological processes, pathogenic processes, or responses to an exposure or intervention” (Califf, 2018).

### 2.2 Study eligibility criteria

Studies with the following characteristics were included: 1) cohort (retrospective and prospective cohort studies), or cross-sectional (observational) studies; in 2) populations with an established PTSD diagnosis (based on clinical diagnostic criteria such as DSM-IV, DSM-5, ICD-10,11); with 3) ‘Cognitive impairment (CI)’ based on a current diagnosis of Mild Cognitive Impairment (MCI), limited neurocognitive disorder or dementia (DSM-IV, DSM-5, ICD-10,11) or neuropsychological assessment criteria, 4) reporting on the association between a specific biomarker (genetic, molecular, neuroimaging or other) and CI in the context of PTSD exclusively. We excluded studies if there were no participants with clinically significant CI, as we were interested in studies where people who had non-impaired cognition were included as control/comparison groups.

### 2.3 Data extraction and quality assessment

After extraction of duplicates four reviewers independently performed study selection based on eligibility criteria (JG, VO, IO, SS), scrutinizing full texts of potentially relevant citations. Data extraction and quality assessment was conducted by two review authors independently (JG, VO, IO, SS). Any discrepancies were resolved by discussion. A standardized data collection form was used extracting information on sample size, country and setting, diagnosis of PTSD, measurement/diagnosis of CI, biomarkers tested, and confounders controlled for (i.e., age, sex, comorbidities). We used the Newcastle-Ottawa Scale (NOS) (Wells, 2000) to assess study quality (see Table 1 Supplementary materials).

## 3 Results

**Fig. 1:**
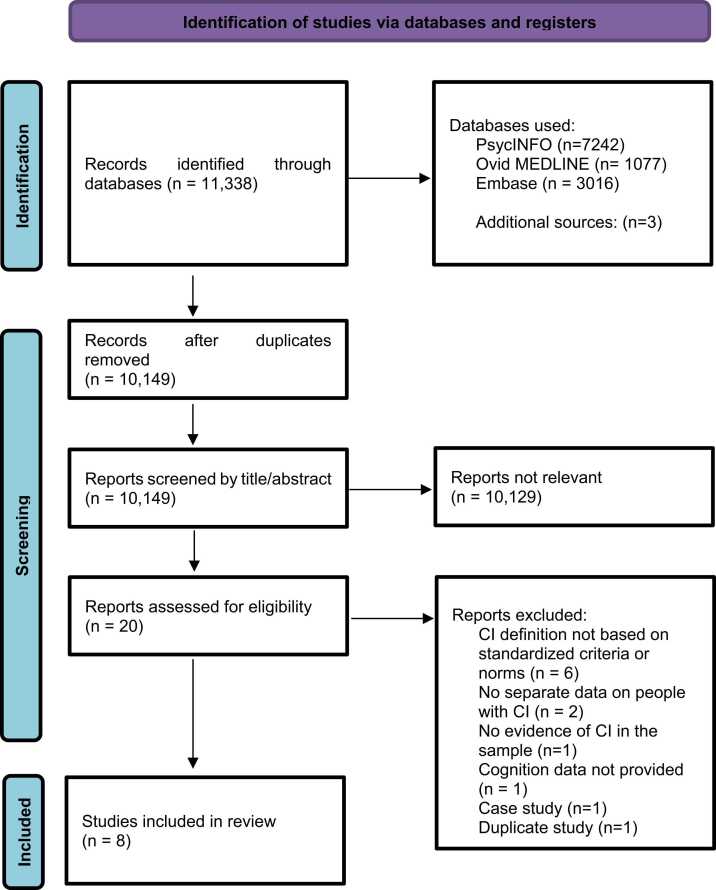
Flow chart of the search strategy.

The flow chart of the search strategy is presented in Fig. 1. A total of 11,338 original articles were retrieved, with a total of 10,149 remaining after removing duplicates. Further screening excluded 10,129 articles by title and abstract leaving 20 articles to be assessed for full text study eligibility. Of these 12 studies were excluded with reasons (see Table 2 Supplementary materials), leaving 8 studies meeting inclusion criteria (Domitrovic Spudic et al., 2022, Easterman et al., 2020, Jagger-Rickels et al., 2022, Kritikos et al., 2022, Kuan et al., 2020, Mohamed et al., 2019, Mohamed et al., 2021, Weiner et al., 2022). Characteristics of the included studies are presented in Table 1.

**Table 1:** Characteristics of included studies.

	Study	Type of biomarker tested	Sample	How MCI/CI was established	Neuropsychological assessment	Biomarker assessed	Adjustment for confounders	Main findings
1	Domitrovic Spudic (2022) Croatia Cross-sectional Psychiatry clinics	**Biochemical**	**Veterans** with combat related PTSD, healthy controls, people with MCI and AD N= 363 (100% male) PTSD = 120 Healthy Controls = 120 MCI = 47 AD = 76 Criteria of PTSD -SCID Mean age: 59.0 (range 50.5-68.5) Where people with dementia excluded? Yes	-Mild cognitive deterioration (MMSE scores 21-25) and Moderate cognitive impairment (MMSE scores 10-20) -Cognitive decline (CDT scores 0-4) -Cognitive decline (MOCA scores 26-30)	1. CDT 2. MMSE 3. MoCA	-Plasma BDNF concentration	-Age -Scores on MMSE, CDT and MOCA	Findings*:* Lower plasma BDNF concentration were found in those with PTSD and CI compared to those with normal cognition
	TRACTS dataset							
2	Esterman (2020) USA Cross-sectional TRACTS dataset	**Functional imaging**	**Veterans** with combat exposure with and without PTSD N = 229 (89% male) PTSD = 140 Controls = 89 Criteria of PTSD - CAPS-IV Mean age: PTSD = 31.31 (SD = 7.71) Controls = 30.91 (SD = 8.30) Where people with dementia excluded? Not reported	CI 1. Verbal memory: -recall (≥2 scores at least 1 SD below normative) -delayed recall 2. Executive functioning and attention: (≥2 scores at least 1 SD below normative)	1. CVLT-II	fMRI: -VAN connectivity	-Age -Gender -Identification -Handedness -Medication status -Head motion	Findings: Lower VAN connectivity in PTSD was found in those with CI in attention, but not in memory and executive function
3	Jagger-Rickels (2022) USA Longitudinal TRACTS dataset	**Functional imaging**	**Veterans** with combat exposure with and without PTSD N = 368 (89% M) N = 314 with imaging PTSD = 181 Controls = 133 PTSD= 121 with imaging follow-up 1-2 years Criteria of PTSD - CAPS-IV Mean age: 31.9, range not reported Where people with dementia excluded? Not reported	CI 1. Verbal memory: -recall (≥2 scores at least 1 SD below normative) -delayed recall 2. Executive functioning and attention: (≥2 scores at least 1 SD below normative)	1. CVLT-II	fMRI: -Functional connectivity	-Age -Gender -Education -WTAR	Findings: Lower functional connectivity between FPCN and learning networks of the limbic system was found in PTSD with CI, especially in those with chronic PTSD at follow-up
	Stony Brook University program							
4	Kritikos (2022) USA Cross-sectional Stony Brook University WTC	**Functional imaging**	**WTC responders** with CI present (+) / absent (-) and PTSD present (+) / absent (-) N = 99 (77.8% male) PTSD+CI+ = 23 PTSD+CI- = 24 PTSD-CI+ =25 PTSD-CI- = 27 Criteria of PTSD -DSM-IV SCID Mean age: PTSD+CI+ =56.1 (SD =5.45) PTSD+CI- = 54.6 (SD = 4.69) PTSD-CI*+* =55.6 (SD =6.24) PTSD-CI- = 57.1 (SD =4.36) Were people with dementia excluded? Yes	CI established based on MoCA≤ 20	1. MoCA	MRI: -Diffusion MRI connectometry analysis	-Age -Gender -Education	Findings: Fractional anisotropy was negatively correlated with CI in PTSD in the fornix, cingulum, forceps minor of the corpus callosum and the right uncinate fasciculus and with PTSD (regardless of CI) in the superior thalamic radiation and the cerebellum
5	Kuan (2020) USA Cross-sectional Stone Brook WTC Health Program	**Biochemical**	Male participants with MCI N = 181 (100% male) PTSD = 39 PTSD-MCI = 34 MCI = 27 Controls = 89 Criteria of PTSD -PCL-17 Mean age = 55.1 (SD = 7.78) Where people with dementia excluded? Yes people with neurodegeneration excluded	MCI established by MoCA	1. MoCA	Proteomics: 276 plasma proteins using the Olink multiplex immunoassay	None	Finding: 16 proteins were associated with PTSD-MCI; 5 proteins were specific to PTSD-MCI comorbidity (compared to PTSD or MCI only)
	ADNI-DOD database							
6	Mohamed (2019) USA Cross-sectional ADNI-DOD	**Functional imaging**	**Veterans** with TBI and/ or PTSD N = 80 (100% male) PTSD = 32 TBI+PTSD = 17 TBI = 10 Control veterans = 21 Criteria of PTSD - CAPS Mean age: PTSD = 70.0 (SD = 2.72) TBI+PTSD = 69.88 (SD = 2.5) TBI = 72.6 (SD = 6.82) Controls = 74.29 (SD = 7.2) Where people with dementia excluded? Yes	MCI established based on ADNI-DOD cognitive test scores	1. ADAS-Cog 2. ANART 3. BNT 4. CDR 5. CDT 6. CFT 7. Ecog 8. MMSE 9. MoCA 10. RAVLT 11. Trials A 12. Trials B	MRI/PET: -tau -Aβ	-Age -APOEε4 -Hypertension -MCI	Finding: CI was more pronounced in PTSD and TBI+PTSD compared to other groups. Compared to controls, all groups showed widespread tau-accumulation in neocortical regions which was associated with CI
7	Mohamed (2021) USA Cross-sectional ADNI-DOD	**Structural imaging** **Functional imaging**	**Veterans** with a history of war-related TBI and/ or PTSD N= 160 (100% male) PTSD = 53 TBI+PTSD = 36 TBI = 23 Control veterans = 48 Criteria of PTSD -DSM-IV Mean age: PTSD = 67.6 (SD = 3.05) TBI+PTSD = 69.1 (SD = 3.84) TBI = 68.8 (SD = 4.69) Controls = 70.8 (SD = 5.59) Where people with dementia excluded? Yes	MCI established based on ADNI-DOD cognitive test scores (CDR ≥ 0.5)	1. ADAS 2. ANART BNT 3. CCT 4. CDR 5. CDT 6. CFT 7. Ecog 8. MMSE 9. MoCA 10. RTMT 11. Trials A 12. Trials B	MRI: - morphometry and white matter alterations PET: -Aβ	-Age -APOEε4 -Hypertension -MCI	Finding: Gray matter atrophy, lower fractional anisotropy and higher diffusivity in major white matter tracts was found in PTSD, and PTSD + TBI compared to controls. Fractional anisotropy and mean diffusivity correlated with CI in PTSD, and PTSD + TBI. In these groups cingulum fractional anisotropy was negatively correlated with amyloid deposits in the posterior cingulate cortex
8	Weiner (2022) USA Longitudinal ADNI dataset	**Structural imaging Functional imaging**	**Veterans** with TBI and/or PTSD and controls N = 289 (99% M) PTSD = 81; TBI = 43; TBI+PTSD = 94; Controls = 71 Criteria of PTSD -using diagnostic codes Mean age: PTSD = 68.2 (SD =3.3) TBI = 70.4 (SD =5.4) TBI+PTSD = 69.8 (SD= 3.1) Controls = 71.4 (SD = 5.8) Where people with dementia excluded? Yes	MCI (clinical assessment based on TICS & EIIDAD)	1. ADAS-Cog13 2. CDR-SB 3. Delayed recall 4. MMSE 5. Trials B	MRI: -Morphological volumes PET: -Aβ -tau	-Age -Education -APOEε4 -Baseline measures	Findings: PTSD and PTSD/TBI CI were not associated with elevated MRI volumes, Aβ or tau

Abbreviations:

Posttraumatic Stress Disorder (PTSD); Mild Cognitive Impairment (MCI); Alzheimer’s disease (AD); Structured Clinical Interview-DSM (SCID); Mini Mental State Examination (MMSE); Clock Drawing Test (CDT); Montreal Cognitive Assessment (MoCA); Brain-Derived Neurotrophic Factor (BDNF); Cognitive Impairment (CI); Translational Research Center for Traumatic Brain Injury and Stress Disorders (TRACTS); Clinical-Administrated PTSD Scale-DSM-IV (CAPS-IV); Standard Deviation (SD); California Verbal Learning Test- II (CVLT-II); Ventral Attention Network (VAN); Wechseler Test of Adult Reading (WTAR); Fronto-Parietal Control Network (FPCN); World Trade Center (WTC); Magnetic Resonance Imaging (MRI); Alzheimer’s Disease Neuroimaging Initiative (ADNI); Post Traumatic Stress Disorder Check List- Specific Version (PCL-17); Alzheimer’s Disease Neuroimaging Initiative-Department of Defense (ADNI-DOD); Traumatic Brain Injury (TBI); Alzheimer’s Disease Assessment Scale- Cognitive subscale 13 (ADAS-Cog 13); American National Adult Reading Test (ANART); Boston Naming Test (BNT); Clinical Dementia Rating-Sum of Boxes (CDR-SB); Clock Copy Test (CCT); Category Fluency Test (CFT); Everyday Cognition (ECog); Rey Auditory Verbal Learning Test (RAVLT); Trial Making Test Part A & B (Trials A & B); Positron Emission Tomography (PET); Amyloid beta (Aβ); Telephone Interview for Cognitive Status 11-item questionnaire (TICS); Eight-Item Interview to Differentiate Ageing and Dementia (EIIDAD)

Of the eight studies meeting our inclusion criteria two were prospective cohort studies following participants up to 5.2 years (Weiner et al., 2022) and 2 years (Jagger-Rickels et al., 2022) respectively (the later study did not report data on the association between CI and PTSD at follow-up), with the remaining six studies being cross-sectional (Domitrovic Spudic et al., 2022, Easterman et al., 2020, Kritikos et al., 2022, Kuan et al., 2020, Mohamed et al., 2019, Mohamed et al., 2021).

### 3.1 Description of cohorts

Domitrovic Spudic 2022 recruited Caucasian male veterans, who participated in the Homeland war in Croatia from 1991 to 1995, with combat related PTSD. Age of onset of PTSD was 40.5 years, and mean age at time of study 59.0 years. Two studies reported data from the Translational Research Center for Traumatic Brain Injury and Stress Disorders (TRACTS) study cohort; Easterman 2020 and Jagger-Rickels 2022; participants were post-9/11 veterans, aged 18–65, who served in Operation Enduring Freedom, Operation Iraqi Freedom, and Operation New Dawn. The sample comprised of combat-exposed veterans with PTSD with a mean age of 31.3 years. Two studies reported data from the Stony Brook University (SBU) program; Kritikos et al., 2022, Kuan et al., 2020 on World Trade Centre responders with a diagnosis of PTSD, mainly residing on Long Island, NY with a mean age of 55.8 years. Three studies reported data from the Alzheimer’s Disease Neuroimaging Initiative Department of Defense cohort (ADNI-DOD); Mohamed et al., 2019, Mohamed et al., 2021, Weiner et al., 2022 which is a multimodal (MRI, PET, and neuropsychological assessment), nonrandomized study. This cohort recruited US Vietnam War veterans with a service-connected traumatic head injury and/or ≥ 1 moderate/severe Traumatic Brain Injury (TBI) or TBI-related diagnostic code and/or ongoing PTSD, with a mean age of 70 years. All three studies additionally reported findings in people with PTSD and TBI.

### 3.2 Samples

Sample sizes ranged from 80 to 368 participants, reporting on a total of 1437 participants, of which 607 were diagnosed with PTSD. Other investigated groups were people with MCI and Alzheimer’s disease (AD). Mean age range of all included groups across studies was 30.9 to 74.3 years. Only 4 studies recruited both male and females (Easterman et al., 2020, Jagger-Rickels et al., 2022, Kritikos et al., 2022, Weiner et al., 2022), with the percentage of females ranging from 1.0 to 22.2% across studies. Most studies recruited white participants with 2 studies not describing ethnicity of the sample (Easterman et al., 2020, Mohamed et al., 2019). Only two studies recruited civilians (Kritikos et al., 2022, Kuan et al., 2020), with the remaining studies recruiting veterans. Only three studies reported on participants’ APOE ε4 status. In Weiner et al. (2022), its presence was reported for a total of 72 individuals (24% of the PTSD group). In Mohamed et al. (2019), only a total of 17 participants were positive for APOE ε4 status (23% of the PTSD group), and in Mohamed et al. (2021) a total of 43 participants (28% of the PTSD group).

### 3.3 Neuroimaging biomarkers

Studies assessed neuroimaging biomarkers using Positron Emission Tomography (PET) (Mohamed et al., 2019, Mohamed et al., 2021, Weiner et al., 2022), and *functional, structural,* and *diffusion* Magnetic Resonance Imaging (MRI) (Easterman et al., 2020, Jagger-Rickels et al., 2022, Kritikos et al., 2022, Mohamed et al., 2021, Weiner et al., 2022). The following biomarkers were investigated:

1) Amyloid-beta (Aβ) burden assessed by Florbetapir (F18-AV-45) (Mohamed et al., 2019, Mohamed et al., 2021, Weiner et al., 2022), 2) Tau protein accumulation assessed by 18 F-flortaucipir (AV-1451) tau PET (Mohamed et al., 2019, Weiner et al., 2022), 3) MRI volumes and morphometry (Jagger-Rickels et al., 2022, Kritikos et al., 2022, Mohamed et al., 2021, Weiner et al., 2022), 4) white-matter microstructural lesions assessed by Diffusion Tensor Imaging (Kritikos et al., 2022; Mohamed et al., 2021), and 5) resting-state functional MRI connectivity (Esterman et al., 2020).

### 3.4 Florbetapir (F18-AV-45) PET

Three studies analyzed Florbetapir (F18-AV-45) PET data, which is used to investigate amyloid-β (Aβ) burden, from the Alzheimer’s Disease Neuroimaging Initiative – Department of Defense USA dataset (ADNI-DoD USA); the prospective study by Weiner et al. (2022) and the cross-sectional studies by Mohamed et al., 2019, Mohamed et al., 2021. In the study by Weiner et al. (2022), there were no significant differences in baseline measures of Florbetapir (F18-AV-45) cortical summary standardized uptake volume rations (SUVR) between groups, including PTSD (p > 0.005). Likewise, no significant differences among groups were reported for Cerebrospinal Fluid (CSF) biomarkers. Follow-up was available for a total of 63 PTSD and 65 PTSD+TBI participants and 60 controls during a total period of 5.2 years. Aβ was described as unchanged but results were not reported. No additional analyses between Aβ, and cognition were conducted.

The cross-sectional study by Mohamed et al. (2019) investigated the correlation between tau accumulation and cerebral total amyloid burden, represented by means of F18-AV-1451 SUVr and F18-AV-45 SUVr voxel-wise maps. The PTSD groups showed positive correlations in the amygdala (r = 0.38, P = 0.03), fusiform gyrus (r = 0.38, P = 0.03), hippocampus (r = 0.36, P = 0.04), inferior temporal gyrus (r = 0.36, P = 0.04), medial temporal gyrus (r = 0.37, P = 0.04), parahippocampus (r = 0.5, P < 0.01), transentorhinal cortex (r = 0.45, P = 0.01), anterior cingulate cortex (r = 0.38, P = 0.03), and posterior cingulate cortex (r = 0.50, P < 0.01).

Mohamed et al. (2021) investigated the correlation between white matter microlesions and amyloid burden in PTSD. Fractional Anisotropy (FA) and Mean Diffusivity (MD) values generated by Diffusion Tensor Imaging (DTI) were correlated with F18-AV-45 SUVr voxel-wise maps. In the PTSD group, a significant negative correlation between F18-AV-45 PET SUVRs in the posterior cingulate cortex and FA values in the cingulum was observed which was not significant in controls (r = - 0.11, p > 0.05).

### 3.5 Flortaucipir (F18-AV-1451) PET

Two studies analyzed Flortaucipir (F18-AV-1451) PET data, which is used to investigate tau accumulation, from the Alzheimer’s Disease Neuroimaging Initiative – Department of Defense USA dataset (ADNI-DoD USA); the prospective cohort by Weiner et al. (2022) and the cross-sectional study by Mohamed et al. (2019). Similarly to Aβ results, Weiner et al. (2022) did not find a significant difference in CSF tau and tau accumulation indicated by flortaucipir SUVR at baseline between PTSD, PTSD+TBI and controls (p > 0.05). Longitudinal assessments of tau were described as unchanged but not reported.

Mohamed et al. (2019) found widespread tau accumulation in the PTSD group, as represented by F18-AV-1451 SUVRs higher means when compared to healthy controls. Reported foci were on the brainstem (1.00 +0.01 vs. 0.95 +0.05, P = 0.03), the precuneus (1.1 +0.07 vs. 1.06 +0.07, P = 0.05), insula (1.07 +0.09 vs. 1.03 +0.06, P = 0.02), the pars-opercularis (1.04 +0.1 vs. 1.0 +0.06, P = 0.04), cuneus (1.10 +0.09 vs. 1.05 +0.06, P = 0.04), pericalcarine (1.10 +0.1 vs. 1.07 +0.07, P = 0.03), superior temporal gyrus (1.04 +0.7 vs. 0.99 +0.07, P = 0.05), transverse temporal gyrus (1.00 +0.08 vs. 0.93 +0.06, P = 0.003), and medial orbitofrontal cortex (1.07 +0.07 vs. 1.02 +0.07, P = 0.04). The PTSD group showed a positive correlation between the ADAS-CoG score and F18-AV-1451 SUVR in the lateral occipital cortex (r = 0.43, P = 0.05). Moreover, a positive correlation between ECoG total score and F18-AV-1451 SUVR was reported for the caudal medial frontal gyrus (r = 0.4, P = 0.02), fusiform gyrus (r-0.35, P = 0.05), lateral occipital cortex (r = 0.40, P = 0.02), inferior temporal gyrus (r = 0.40, P = 0.02), postcentral gyrus (r = 0.40, P = 0.02), precentral gyrus (r = 0.41, P = 0.02), posterior cingulate cortex (r = 0.38, P = 0,03), anterior cingulate cortex (r = 0.45, P = 0.01), superior parietal lobe (0.41, P = 0.02), and cuneus (r = 0.47, P = 0.01). Finally, the PTSD group showed a trend towards a positive correlation between CDR scores and F18-AV-1451 SUVR.

### 3.6 Neuroimaging, structural biomarkers

Two studies analyzed MRI data, which is used to investigate morphological changes in brain volume, from the Alzheimer’s Disease Neuroimaging Initiative – Department of Defense USA (ADNI-DoD USA) dataset. There were no baseline differences between groups in volumes of hippocampus (p > 0.59), amygdala (p > 0.58), enthorhinal cortex (p > 0.14), temporal cortex (p > 0.99), parietal cortex (p > 0.98), or white matter hyperintensities volume, (p > 0.68) in the prospective cohort of Weiner et al. (2022). Furthermore, no significant decline in volumes were observed in the PTSD group during follow-up analyses, and the differences in annual rates of changes across groups did not reach significance (P > 0.7). Importantly, a subgroup analysis comparing the ensemble of MCI participants distributed across all study groups, including PTSD, and the cohort of male MCI patients from the National Institutes of Health (NIH)-funded ADNI study showed that the MCI participants (n = 33) recruited in Weiner et al. (2022) had higher hippocampal volumes than their NIH (n = 248) counterparts (0.50 +0.07% intracranial volume vs. 0.46 +0.08).

Mohamed et al. (2021) reported mean reduced total brain volume in those with PTSD (1034.7 D 92.1 cm^3^, effect size= 1.77, p = 0.007) compared to controls (1192.6 SD 86.2 cm^3^). Greater gray matter atrophy in PTSD was found in the superior frontal gyrus, superior and middle occipital gyrus, right hippocampus, right amygdala, supramarginal gyrus and angular gyrus (P < 0.01), but no association was observed between these morphological changes and CI.

### 3.7 Neuroimaging, white matter microstructural lesions

The cross-sectional study by Mohamed et al. (2021) using Diffusion Tensor Imaging (DTI), found several significant negative and positive associations between different measures of cognition (Boston Naming Test, MOCA and MMSE) and *Fractional Anisotrophy (FA),* and *Mean Diffusivity* (MD) in the PTSD group. In the PTSD group positive correlations were reported between BNT scores and FA in bilateral Inferior Longitudinal Fasciculus (ILF), right Superior Longitudinal Fasciculus (SLF) and tapetum. By contrast, negative correlations between BNT scores and MD were observed in the SLF, arcuate fasciculus, Inferior Fronto-Occipital Fasciculus (IFO), tapetum, anterior ILF and Internal Capsule (IC). Additionally, a negative correlation between MOCA scores and FA in SLF and the tapetum were observed for the PTSD group, whilst no significant association was observed with MD in any group. In the PTSD group, MMSE and MD measures were negatively correlated in the SLF, ILF and IFO in the PTSD group.

In the cross-sectional study by Kritikos et al. (2022) reporting on WTC survivors, which also used DTI, a region-of-interest analysis showed patterns of reduced white matter FA highly overlapping in both PTSD and PTSD with cognitive impairment groups. These included the bilateral fornix, the left cingulum, the forceps minor of the corpus callosum, the right uncinate fasciculus, and the bilateral cerebrum (PTSD T = 2.5, FDR=0.0322; PTSD+CI T = 2.5, FDR=0.0132). Additionally, the PTSD group without cognitive impairment exhibited reduced white matter FA in the right superior thalamic radiation and the right ILF (T = 2.5, FDR=0.0322).

### 3.8 Neuroimaging, resting-state fMRI

Esterman et al. (2022) used rs-fMRI to assess the resting-state connectivity within the Ventral Attention Network (VAN), comprising regions in the ventral prefrontal cortex (vPFC) and the temporoparietal junction (TPJ), in patients diagnosed with PTSD presenting with clinically significant cognitive impairment. The authors reported a significant decrease in resting-state connectivity within VAN for the PTSD group exhibiting clinically significant attentional impairment (χ^2^ =8.62, P = 0.013).

In the longitudinal study by Jagger-Rickels et al. (2022), a subgroup analysis comparing PTSD patients with clinically significant impairment in memory and attention, PTSD patients with average performance, and PTSD patients with above-average performance in these domains, showed differential patterns of frontoparietal control network (FPCN) resting-state connectivity with the limbic network (LN), which predicted PTSD chronicity and worsening of symptoms in a two-year follow-up period. Specifically, PTSD patients with clinically significant impairment exhibited a decreased resting-state connectivity among the FPCN and LN networks, which correlated with the chronicity and worsening of PTSD symptoms in the two-year follow-up period, whereas PTSD patients with above-average performance did not exhibit any alteration in connectivity among these networks, and showed a decrease in symptom severity.

### 3.9 Biochemical biomarkers

Two cross-sectional studies assessed protein biomarkers; one study included a set of proteomics in which 276 plasma proteins were assessed using the Olink multiplex immunoassay (Kuan et al., 2020); and a second study assessing plasma Brain Derived Neutrophic Factor (BDNF) levels (Domitrovic Spudic et al., 2022).

### 3.10 Proteomics

In the study by Kuan (2020) a total of 16 plasma proteins were identified to be upregulated in those with PTSD and comorbid MCI (n = 34) compared to healthy controls (n = 81) (p < 0.05). Six proteins in total stayed significant at False Discovery Rate (FDR) < 0.1; Neurocan core protein (NCAN), Brevican core protein (BCAN), Cathepsin S (CTSS), Macrophage Scavenger Receptor types I and II (MSR1), MAM domain-containing glycosylphosphatidylinositol anchor protein 1 (MDGA1), and Carboxypeptidase A2 (CPA2). All these proteins, except NCAN, were associated with disease burden of PTSD co-occurring with MCI (using BIC models).

### 3.11 Brain derived neurotrophic factor (BDNF)

In the study by Domitrovic Spudic et al. (2022), plasma BDNF was significantly lower in PTSD, MCI and AD compared to controls (F=40.22; df=3; p < 0.001). Lower levels of BDNF concentration were found in those with PTSD and cognitive impairment compared to those with normal cognition in the PTSD group (p < 0.001).

### 3.12 Quality of studies

Table 3 (see Supplementary materials) provides an overview of quality assessment for all studies meeting inclusion criteria using the NOS scale. Overall study quality was fair to low given that most studies reported on small samples. Low study quality was also observed in relation to representative cohorts, confounders being adjusted and the lack of prospective data, limiting conclusions of the utility of each of the biomarkers tested.

## 4 Discussion

To the best of our knowledge, this is the first systematic review investigating biomarkers associated with CI in PTSD. We aimed to outline current evidence on the different biomarkers used to detect and track CI in PTSD populations. We found insufficient evidence to support the use of any biomarker to measure CI in this group. Our review identifies that the current literature is scarce but is nevertheless increasing in the last few years with a total of 8 studies meeting our inclusion criteria. Of these, a total of 6 studies focused on neuroimaging biomarkers; three PET studies (Aβ, tau) (Mohamed et al., 2019, Mohamed et al., 2021, Weiner et al., 2022), five functional, anatomical and diffusion MRI studies (Easterman et al., 2020, Jagger-Rickels et al., 2022, Kritikos et al., 2022, Mohamed et al., 2021, Weiner et al., 2022), and two studies investigating biochemical biomarkers (BDNF and several proteomics) (Domitrovic Spudic et al., 2022, Kuan et al., 2020). Overall, studies were rated as low to fair quality, with designs being mainly cross-sectional which limits any conclusions around causality. An important limitation of the current evidence base is that most studies to date report on data derived from the same cohorts (TRACTS and ADNI-DOD cohort; Stony Brook University program).

### 4.1 Summary of main findings

We found that neuroimaging studies overall showed significant cross-sectional associations between CI and PTSD. Specifically, DTI FA and MD values (Kritikos et al., 2022, Mohamed et al., 2019, Mohamed et al., 2021), VAN resting-state connectivity (Esterman et al., 2020) and FPCN-LN resting-state connectivity (Jagger-Rickels et al., 2022) were all associated with CI (established by BNT, MOCA, ECoG, ADAS-CoG, and a composite measure of attention including TOVA and DSP) in this group. On the other hand, data from a single longitudinal study found that MRI volumes were not predictive of CI in PTSD (Weiner et al., 2022).

In the longitudinal study by Weiner et al. (2022) no differences were observed in the baseline volumes of hippocampal, amygdala, enthorhinal cortex, temporal cortex, and parietal cortex, between those with CI and PTSD, and controls (Weiner et al., 2022). The same also applied for presence of white matter hyperintensities (Weiner et al., 2022). In the cross-sectional study by Mohamed et al. (2021), mean reduced total brain volume and greater gray matter atrophy in PTSD were also not associated with CI. These findings are in line with prior studies (Elias et al., 2020) reporting no differences in brain volumes of older PTSD populations (mean age 67.80 years), compared to aged-matched controls (mean age 70.23 years).

The results of PET studies on Aβ and tau protein are primarily based on a small set of studies using data from the same cohort (ADNI-DOD). Among these, one found an association between Aβ and tau protein, and white matter cortical Aβ depositions and CI in PTSD (Mohamed et al., 2021), whereas the other did not (Weiner et al., 2022). Although Mohamed et al. (2019) did report higher cortical Aβ ([^18^F]-AV45 PET SUVR) in several brain areas of PTSD exposed groups with higher prevalence of MCI, the authors did not compare these findings with controls or examine whether these biomarkers were associated with CI.

Similarly in one cross-sectional study tau accumulation in neocortical regions was higher in PTSD exposed groups and significantly associated with CI (Mohamed et al., 2019), whereas longitudinal follow-up data from the same cohort (Weiner et al., 2022) found no significant differences in tau accumulation between groups; the two studies used data of the same cohort which were older veterans with PTSD; but were different on study design (cross-sectional versus prospective cohort), making comparison of results difficult. Given inconsistent findings, and limitations in terms of study design the contribution of possible amyloidosis or tauopathy involved in PTSD and associated CI remains inconclusive.

The studies included in our systematic review studied different age groups of people with PTSD, from young people (Domitrovic Spudic et al., 2022), to older populations (Weiner et al., 2022). Future studies should justify their inclusion criteria and should minimize exclusion criteria to avoid limiting the generalizability of their results across PTSD populations. No studies included a power calculation and the small number of participants in studies is of concern. As studies are small it becomes increasingly likely that potentially significant associations will not be detected; this additionally limits the number of variables that can be included in multivariate analyses increasing the risk of spurious findings. In an heterogeneous disorder such as PTSD, small sample sizes mean the cohorts are unlikely to be representative of all patients with PTSD.

Our review identified several plasma proteins positively associated with the comorbidity of PTSD and CI and MCI: these included BVAN, CTSS, MSR1, MDGA1 and CPA2 (Kuan et al., 2020). These proteins are implicated in neuronal and synaptic processes, as well as neuroinflammatory processes which are known to contribute to the etiopathology of AD and other neurodegenerative diseases. BDNF levels, a protective factor against future occurrence of dementia and AD, implicated in neuronal and synaptic processes taking place in the neocortex and the hippocampus, were overall lower in the context of CI in PTSD, however this was independent of CI status (Domitrovic Spudic et al., 2022). These data, although preliminary, indicate that BDNF may be an important biomarker for detecting CI in PTSD (Guo et al., 2019). It will be important that future studies replicate these results given that proteomics and BDNF, are not validated biomarkers in dementia diagnostics (Li et al., 2022). Large scale studies replicating the results of Kuan et al. (2020) in several prognostic protein markers such as CTSS, are important given that plasma proteins may be more cost-effective and less burdensome for patients compared to imaging methods.

Most studies identified to date used simplistic statistical analyses and many did not examine the influence of confounders. Future longitudinal cohort studies will be important in elucidating the association between CI in PTSD and specific biomarkers by investigating multiple mediating factors including APOE ε4 (Averill et al., 2019, Elias et al., 2020), psychiatric morbidity (Qassem et al., 2021), such as depression and intake of antidepressants, or other medical comorbidities such as cardiovascular disorders that could contribute to risk of dementia (Jacob et al., 2018). TBI status which was assessed and examined in several studies remains an important confounder, given evidence that it is associated with elevated risk of dementia, accumulation of beta-amyloid and tau protein (Graham and Sharp, 2019). Future work should also test how variations in sex, socio-economic status, and ongoing stressors (e.g., unemployment, financial adversity) (Gunak et al., 2020), or inconsistencies in methodological practices such as sample collection, assaying, and data cleaning, may contribute to variations in results.

Despite the originality of our review there are significant limitations. This relates to the overall small evidence base and significant risk of bias across studies. Many studies identified by our review were cross-sectional with very few prospective cohort studies. The majority of studies were also reporting on veteran populations. Given therefore the small number of studies in civilians it will be important that future work in the area includes groups across several trauma exposed populations, particularly women who remain under-represented across both veteran and civilians while at the same time being at higher risk of PTSD (Olff, 2017).

Quality ratings indicated that only a small number of studies controlled for important confounding factors such as APOE status, which is a strong genetic risk factor for dementia (Gharbi-Meliani et al., 2021, Neu et al., 2017). Other risk factors not consistently controlled for included age, education, and medication use with no studies to date controlling for several confounding factors simultaneously. Thus, findings remain significantly limited given that other factors that could explain the association between several biomarkers and CI in PTSD were not controlled for. Future studies should accommodate for the heterogenous nature of PTSD including the dissociative subtype of PTSD (van Huijstee and Vermetten, 2018) as well as controlling for several other pathologies such as TBI associated encephalopathy (McKee et al., 2013), and dementia subtype (Bonanni et al., 2018). Despite similarities across studies cognitive impairment definitions differed (Jak et al., 2009, Riley et al., 2019). Future studies should therefore aim to harmonise cognitive measures used to detect CI so that comparison across studies can be made.

## 5 Conclusion

This is the first systematic review investigating biomarkers associated with CI in PTSD. Findings from a small evidence base indicate that functional, anatomical and diffusion neuroimaging, specifically white matter microstructural lesions, and resting-state functional connectivity are cross-sectionally associated with CI in PTSD. Findings on Aβ and tau protein remain preliminary and inconsistent. The contribution of biochemical biomarkers such as BDNF and other core proteins (BAN, CTSS, MSR1, MDGA1 and CPA2) in the association between CI and PTSD require further investigation.

Our review has highlighted the important methodological, and statistical limitations of studies examining biomarkers of CI in PTSD. It is clear that continuing to publish small cross-sectional studies in this area is unlikely to inform the future use of biomarkers for CI progression in PTSD. Large scale multidisciplinary longitudinal follow-up studies are needed to identify which specific biomarkers may predict or diagnose CI and dementia in PTSD.

## Author contributions

Junling Guo: conceptualisation of the review; selection of studies; data extraction; data analysis; data quality; writing, review and editing of manuscript. Vasiliki Orgeta: conceptualisation of the review; selection of studies; data extraction; data analysis; data quality; writing, review and editing of manuscript. Isadora Olivé: selection of studies; review and editing of final draft. Erik Hoff: biomarkers expertise; review and editing of final draft. Jonathan Huntley: biomarkers expertise; review and editing of final draft. Miranda Olff: PTSD and biomarkers expertise; review and editing of final draft. Sjacko Sobczak: conceptualisation of the review; selection of studies; data extraction; data analysis; data quality; writing, review and editing of manuscript.

## Declaration of Competing Interest

All authors have nothing to declare. The authors have no conflicts of interest to report.

## Data Availability

No data was used for the research described in the article.
