# Supplementary material for: Biomarkers associated with cognitive impairment in post-traumatic stress disorder: A systematic review of current evidence
Source: Ageing Res Rev. 2024 Mar;95:102198. doi: 10.1016/j.arr.2024.102198 (PMC11932974; doi:10.1016/j.arr.2024.102198)
Supplement: Supplementary file 1 — Supplementary material. [file mmc1.docx]

Search Strategy of the Review for PsyInfo

1. Dement*.mp.

2. Alzheime*.mp.

3. AD.mp.

4. "Lewy Body".mp.

5. LBD.mp.

6. FTD*.mp.

7. FTLD.mp.

8. "cognitive decline".mp.

9. "cognitive impairment".mp.

10. delirium.mp.

11. deliri*.mp.

12. Wernicke Encephalopathy.mp.

13. MCI.mp.

14. (chronic adj cerebrovascular).mp.

15. ("organic brain disease" or "organic brain syndrome").mp. [mp=title, abstract, heading word, table of contents, key concepts, original title, tests & measures, mesh]

16. ("normal pressure hydrocephalus" and "shunt*").mp.

17. "benign senescent forgetfulness".mp.

18. (cerebr* adj2 deteriorat*).mp.

19. (cerebral* adj2 insufficient*).mp.

20. (pick* adj2 disease).mp.

21. (creutzfeldt or jcd or cjd).mp.

22. huntington*.mp.

23. binswanger*.mp.

24. korsako*.mp.

25. "preclinical AD".mp.

26. "pre-clinical AD".mp.

27. ("preclinical alzheimer*" or "pre-clinical alzheimer*").mp.

28. ACMI.ti,ab.

29. exp Alzheimer's Disease/Dementia/Cognitive Impairment/Dementia with Lewy Bodies/Delirium

30. PTSD.mp.

31. post?traumatic*.mp.

32. "Stress Disorder".mp.

33. flashback*.mp.

34. disaster*.mp.

35. victim*.mp.

36. "stress disorder*".mp.

37. "acute stress".mp.

38. Posttraumatic Stress Disorder/Military Veterans/Child Abuse/Sexual Abuse/War/Disasters/Natural Disasters/Crime Victims/Victimization/Intimate Partner Violence/Rape/ Sex Offenses/ Domestic Violence/ Stress Reactions

39. biomarker*.mp.

40. genetic*.mp.

41. epigenetic*.mp.

42. gene*.mp.

43. polymorphism*.mp.

44. "single nucleotide polymorphism".mp.

45. "single nucleotide polymorphisms".mp.

46. mutation*.mp.

47. methylation*.mp.

48. neural.mp.

49. neuronal.mp.

50. structural.mp.

51. autonomic.mp.

52. physiolog*.mp

53. "skin conductance".mp.

54. "galvanic skin response".mp.

55. "galvanic skin responses".mp.

56. "heart rate".mp.

57. startle.mp.

58. "defense mechanism".mp.

59. "defense mechanisms".mp.

60. "defensive reactivity".mp.

61. predict*.mp.

62. "Neurobiological marker*".mp.

63. neuroanatomical.mp.

64. amygdala.mp.

65. "prefrontal cortex".mp.

66. PFC.mp.

67. hippocamp*.mp.

68. neuroendocrine.mp.

69. HPA-Axis.mp.

70. "Glucocorticoid negative feedback".mp.

71. "baseline cortisol".mp.

72. Pituitary adenylate cyclase-activating polypeptide.mp.

73. PACAP.mp.

74. Steroid hormones.mp.

75. estradiol.mp.

76. allopregnanolone.mp.

77. dehydroepiandrosterone.mp.

78. testosterone.mp.

79. metabolic hormones.mp.

80. NPY.mp.

81. Ghrelin.mp.

82. Insulin.mp.

83. Endocannabinoids.mp.

84. neuroimaging.mp.

85. "brain morphology".mp.

86. "brain volume".mp.

87. neurofunctional.mp.

88. "functional magnetic resonance imaging".mp.

89. "positron emission tomography".mp.

90. "single-photon emisson computerized tomography".mp.

91. "near-infrared spectroscopy".mp.

92. electroencephalography.mp.

93. fMRI.mp.

94. MRI.mp.

95. PET.mp.

96. SPECT.mp.

97. NIRS.mp.

98. EEG.mp.

99. cortisol.mp.

100. neurotrophin*.mp.

101. "brain derived neurotrophic factor".mp.

102. BDNF.mp.

103. "arginine vasopressin".mp.

104. AVP.mp.

105. oxytocin.mp.

106. "noradrenergic dysregulation".mp.

107. catecholamine*.mp.

108. progesterone.mp.

109. "cornu ammonis ".mp.

110. CAmp.

111. "dentate gyrus".mp.

112. "locus coeruleus".mp.

113. "neurobiological models".mp.

114. "cognitive behav* models".mp.

115. "machine learning".mp.

116. "gaussian process".mp.

117. "support vector".mp.

118. "random forest".mp.

119. "decision tree".mp.

120. "decision trees".mp.

121. "support vector machine".mp.

122. "support vector machines".mp.

123. "multivariate pattern analysis".mp.

124. "multivariate pattern analyses".mp.

125. "gaussian process classifier".mp.

126. "gaussian process classifiers".mp.

127. SVM.mp.

128. MVPA.mp.

129. GPC.mp.

130. "pattern recognition".mp.

131. exp Biological Markers/ Risk Factors/ Proteins/ Genetic Models/ Genetic Transcription/ Genetic Translation/ Epigenetics/ DNA/ Genetics/Genes/ Gene Expression/ Stress/Neural Plasticity/ Environment/ Phenotypes/ Etiology/ Gene Expression/ Polymorphism/ Serotonin/ Genotypes/ Nucleotides/ Neural Receptors/ Genome/ Mutations/ Biological Neural Networks/ Neural Development/ Neural Lesions/ Neural Networks/ Neural Pathways/ Neural Regeneration/ Neurons/ Autonomic Nervous System/ Hypothalamus/ Vasopressin/ Biology/Skin Resistance/ Galvanic Skin Response/ Psychophysiology/ Heart Rate/Emotions/ Physiological Correlates/ Childhood Development/ Emotional Responses/Emotional States/ Defense Mechanisms/ Defensiveness/ Exposure/ Prediction/ Neurobiology/ Neuropsychology/ Neuroanatomy/ Brain/ Gray Matter/ Brain Size/ Cingulate Cortex/ Amygdala/ Prefrontal Cortex/ Medial Prefrontal Cortex/ Hippocampus/ Neuroendocrinology/ Hypothalamic Pituitary Adrenal Axis/ Glucocorticoids/ Corticotropin/ Dexamethasone/ Testosterone/ Estradiol/ Neuropeptide Y/ Ghrelin/ Insulin/ Neuroimaging/ Functional Magnetic Resonance Imaging/ Neuroimaging/ Positron Emission Tomography/ Electroencephalography/ Magnetic Resonance Imaging/ Single Photon Emission Computed Tomography/ Neurotrophic Factor/ Brain Derived Neurotrophic Factor/ Oxytocin/ Catecholamines/ Progesterone/ Locus Ceruleus/ Machine Learning/ Pattern Recognition (Cognitive Process)

Table 1 Modified Newcastle-Ottawa Scoring scale assessing study quality

| **Selection** |
| --- |
| 1. Representativeness of the exposed cohort |
| a) truly representative (1 point **🟑**)  b) somewhat representative (1 point **🟑**)  c) selected group (0 points)  d) no description of the derivation of the cohort (0 points) |
| 1. Selection of the non exposed cohort |
| a) drawn from the same community as the exposed cohort (1 point **🟑**)  b) drawn from a different source (0 points)  c) no description (0 points) |
| 1. Ascertainment of exposure |
| a) secure record (1 point **🟑**)  b) structured interview (1 point **🟑**)  c) written self-report (0 points)  d) no description (0 points) |
| 1. Demonstration that outcome of interest was not present at start of study |
| a) yes (1 point**🟑**)  b) no (0 points) |
| **Comparability** |
| 1. Comparability of cohorts on the basis of the design or analysis |
| a) study controls for most important factor (1 point **🟑**)¹  b) study controls for any additional factor (1 point **🟑**) |
| **Outcome** |
| 1. Assessment of outcome |
| a) independent blind assessment (1 point **🟑**)  b) record linkage (1 point **🟑**)  c) self-report (0 points)  d) no description (0 points) |
| 1. Was follow-up long enough for outcome to occur |
| a) yes (1 point **🟑**)  b) no (0 points) |
| 1. Adequacy of follow-up of cohorts |
| a) complete follow-up – all participants accounted for (1 point **🟑**)  b) participants lost to follow-up unlikely to introduce bias (1 point **🟑**)  c) follow-up rate less than 80% and no description of those lost (0 points)  d) no statement (0 points) |

*Note:* * = 1 point; ¹we considered age as the most important confounder

Table 2 Excluded studies with reasons

| Study | Reasons of exclusion |
| --- | --- |
| Deri (2021) | Reported data on people with and without CI; no separate data provided |
| Elias (2020) | No evidence of cognitive impairment in the sample |
| Friedman (2019) | CI defined by performance on cognitive measures not based on standardized criteria or norms |
| Jagger-Rickels (2021) | Duplicate study with Jagger-Rickels (2022) |
| Rutherford (2018) | CI defined by performance on cognitive measures not based on standardized criteria or norms |
| Cummings (2016) | People with MCI excluded at baseline; cognition not measured |
| Iacono (2020) | Study reporting on four case studies of veterans with early-onset dementia |
| Ogoh (2018) | CI defined by performance on cognitive measures not based on standardized criteria or norms |
| Guo (2020) | CI defined by performance on cognitive measures not based on standardized criteria or norms |
| Averill (2019) | CI defined by performance on cognitive measures not based on standardized criteria or norms |
| Erjavec (2021) | CI defined by performance on cognitive measures not based on standardized criteria or norms |
| Guo (2019) | Reported data on people with and without CI; no separate data provided |

Table 3 Methodological quality of included studies

|  | **Selection** |  |  |  | **Comparability** |  | **Outcome** |  |  | **Overall Quality** |
| --- | --- | --- | --- | --- | --- | --- | --- | --- | --- | --- |
|  | Representativeness | Non-exposed cohort | Ascertainment of Exposure | Outcome not at baseline | Adjusted for important factor | Adjusted for additional factor | Assessment of outcome | Follow-up | Adequacy of follow-up |  |
| Domitrovic Spudic 2022 | 0 | 0 | 1 | 1 | 1 | 1 | 0 | 0 | 0 | Low |
| TRACTS dataset studies | | | | | | | | | | |
| Esterman 2020 | 0 | 0 | 1 | 0 | 1 | 1 | 0 | 0 | 0 | Low |
| Jagger-Rickels 2022 | 0 | 0 | 1 | 0 | 1 | 1 | 1 | 1 | 0 | Fair |
| Stony Brook University program studies | | | | | | | | | | |
| Kritikos 2022 | 1 | 1 | 1 | 1 | 1 | 1 | 0 | 0 | 0 | Fair |
| Kuan 2020 | 1 | 1 | 0 | 1 | 0 | 0 | 1 | 0 | 0 | Low |
| ADNI-DOD database studies | | | | | | | | | | |
| Mohamed 2019 | 0 | 1 | 1 | 1 | 1 | 1 | 0 | 0 | 0 | Fair |
| Mohamed 2021 | 0 | 1 | 1 | 1 | 1 | 1 | 0 | 0 | 0 | Fair |
| Weiner 2022 | 0 | 1 | 1 | 1 | 1 | 1 | 1 | 1 | 1 | Good |
